# Factors Associated With Treatment Delay in Breast Cancer: A Prospective Study

**DOI:** 10.7759/cureus.13242

**Published:** 2021-02-09

**Authors:** Raja Rahool, Ghulam Haider, Muhammad Hayat, Mehwish R Shaikh, Paras Memon, Bhunisha Pawan, Kiran Abbas

**Affiliations:** 1 Oncology, Jinnah Postgraduate Medical Centre, Karachi, PAK; 2 Medicine, Jinnah Postgraduate Medical Centre, Karachi, PAK

**Keywords:** breast neoplasms, breast carcinoma, financial stress, fear, delayed treatment, time-to-treatment, patient care, breast tumors

## Abstract

Introduction

The frequency of breast cancer (BC) is increasing among Pakistani females. It has been estimated that one out every nine women is predicted to develop BC, which is the highest disease occurring rate in the Asian population. The study aimed to evaluate the factors responsible for delay in diagnosis of BC in Sindh, Pakistan.

Methodology

This study was conducted at the Medical Oncology Department of Jinnah Postgraduate Medical Center Karachi from December 2018 to June 2019. All women between 17 and 80 years diagnosed with BC who had treatment delay of more than six months were included in the study using a non-probability consecutive sampling technique. The face-to-face interviews were conducted by the researcher himself and all the data regarding demographics and factors related to treatment delay of BC was noted in a structured questionnaire. Data were analyzed using SPSS version 23 (IBM Corp., Armonk, NY).

Results

Appointment delay was significantly associated with a treatment-seeking delay in patients (p=0.03). Lack of awareness was another significant factor associated with treatment delay in BC patients. About 50 (70.4%) women who reported a lack of awareness sought treatment after 10 months of their first onset of symptoms (p=0.001). Cultural beliefs were a significant cause of treatment delay of 10-12 months in 71.8% of patients (p=0.021). Financial constraints significantly correlated with treatment delay (p=0.015).

Conclusion

The cultural beliefs, poor financial status, and lack of awareness are the significant factors for the treatment delay in BC patients. Promoting female health awareness can tackle many of these issues.

## Introduction

Breast cancer (BC) is the commonest cancer, a major global health issue, and a leading cause of death among females. The occurrence rate of BC is rising globally, but the survival rate is better in developed countries due to diagnosis at an early stage and advanced treatment [1,2]. About 1,000,000 females are yearly diagnosed having BC [2].

The frequency of BC is increasing among Pakistani females. It has been estimated that one out every nine women is predicted to develop BC, which is the highest disease occurring rate in the Asian population [2,3]. People of all ages are influenced by cancer and it is creating a huge social and financial burden on the residents of Pakistan [2]. Most of the areas of Pakistan are underprivileged and the majority of the population resides in rural areas [2]. BC accounts for 32% of all tumors in women of Karachi [4].

The chances of improvement, cure, quality of life, and survival are highly related to early diagnosis and detection of BC and onset of treatment. A delay in diagnosis may lead to advanced stages of tumor [5,6]. It is speculated that in Pakistan, social-cultural concerns, improper access to healthcare facilities, unavailability of diagnostic tools, illiteracy or low education, lack of awareness about signs and symptoms of BC, low socioeconomic status, and use of traditional therapy and false beliefs among females could lead to treatment delay resulting in poorer prognosis at presentation [7-9].

More than half of the females presented late with an advanced stage of BC, i.e., 3 or 4 in Pakistan [10]. The delay in treatment of BC after the appearance of signs and symptoms can be decreased by identifying potential factors related to the delay. Therefore, the present research was carried out to identify the risk factors responsible for treatment delay of patients with BC. This research would be helpful for the early management and treatment of patients with BC, promotion of health education among females, improved survival rates, and better prognosis of BC patients.

## Materials and methods

A prospective observational study was conducted at the Medical Oncology Department of Jinnah Medical Postgraduate Center from December 10, 2018, to June 10, 2019, and it was an observational analytical study. The sample size was estimated using Open epi online sample size calculator by taking the frequency of factor associated with treatment delay of BC, i.e., negative physical breast examination as 24.4% [11], the margin of error as 6%, and 95% confidence level, the calculated sample size came out as 197. All the females of age 17-80 years diagnosed with BC who had treatment delay of more than 6 months were included in the study using a non-probability consecutive sampling technique. Women with psychiatric problems and who did not give consent were excluded from the study.

After taking approval from the ethical review committee data was collected. Informed written or verbal consent was taken from all the eligible patients. The face-to-face interviews were conducted by the researcher himself and all the data regarding demographics and factors related to treatment delay of BC was noted in a structured questionnaire. 

Data was entered and analyzed using SPSS version 23 (IBM Corp., Armonk, NY). Quantitative variables were presented as mean and SD whereas qualitative variables were presented as frequency and percentage. One-way ANOVA was applied to address the delay time with factors related to treatment delay. A p-value ≤ of 0.05 was taken as statistically significant.

## Results

A total of 197 patients were included in the study. The age of the patients was 45.38±11.58 years. The mean BMI was reported as 26.28±5.49 kg/m^2^. The majority of the patients were living in urban areas (n=139, 70.6%) whereas 58 patients (29.4%) were living in rural areas. Most of them were illiterate (n=103, 52.3%), belonged from low class (n=149, 75.6%), housewives (n=168, 85.3%) and married (n=126, 64%) as shown in Table 1.

**Table 1:** Sociodemographic characteristics of the study population.

Characteristics	Mean ± SD
Age (years)	45.38±11.58
BMI (kg/m^2^)	26.28±5.49
	n (%)
Residence	
Urban	139 (70.6)
Rural/metropolitan	58 (29.4)
Education	
Illiterate	103 (52.3)
Matric	14 (7.1)
Primary	39 (19.8)
Intermediate	30 (15.2)
Graduate	10 (5.1)
Postgraduate	1 (0.5)
Socioeconomic status	
Low class (income: <15,000 rupees)	149 (75.6)
Middle class (income: 15,000-30,000 rupees)	33 (16.8)
High class (income: >30,000 rupees)	15 (7.6)
Occupation	
Student	5 (2.5)
Housewife	168 (85.3)
Employed	23 (11.7)
Unemployed	1 (0.5)
Marital Status	
Married	126 (64)
Separated	15 (7.6)
Single	11 (5.6)
Widow	45 (22.8)

**Figure 1:**
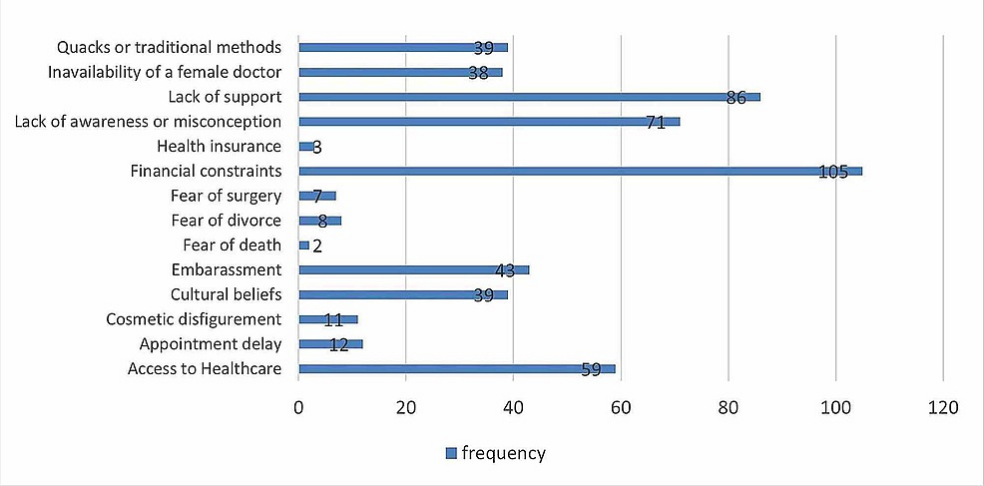
Factors associated with treatment delay in patients with breast cancer.

According to factors associated with treatment delay, almost more than half of the patients had financial constraints 105 (53.3%). 86 (43.7%) women informed us about the lack of support (like moral, financial, and social support). 71 (36.0%) women had a lack of awareness or misconception regarding treatment and 59 (29.9%) women had treatment delay due to inaccessibility to healthcare. About 43 (21.8%) women had fears of social embarrassment regarding the treatment of disease, 39 (19.8%) did not seek treatment due to cultural beliefs, and 38 (19.3%) women had avoided treatment due to the unavailability of a female doctor. Approximately 39 (19.8%) females were presented late due to prior use of traditional methods such as unconventional and herbal therapy. Twelve women (6.1%) had appointment delays; 11 (5.6%) women had concerns regarding cosmetic disfigurement as described in Figure 1. 

Appointment delay was significantly associated with the treatment-seeking delay in patients (p=0.03). Lack of awareness was another significant factor associated with treatment delay in BC patients. About 50 (70.4%) women who reported a lack of awareness sought treatment after 10 months of their first onset of symptoms (p=0.001). Cultural beliefs were a significant cause of treatment delay of 10-12 months in 71.8% of patients (p=0.021). Financial constraints significantly correlated with treatment delay (p=0.015). Over one-half of the patients with financial constraints had treatment delays of more than nine months. See Table 2 for details.

**Table 2:** Factors related to treatment in association with duration of delay.

Factors	Duration of delay		p-value
	6-9 months (n=91)	10-12 months (n=106)	
Traditional methods			
Yes	16(47.1%)	18(52.9%)	0.911
No	75(46%)	88(54%)	
Appointment delay			
Yes	3(25.0%)	9(75.0%)	0.03
No	105(56.8%)	80(43.2%)	
Lack of awareness			
Yes	21(29.6%)	50(70.4%)	0.001
No	66(33.5%)	60(30.4%)	
Fear of surgery			
Yes	3(42.9%)	4(57.1%)	0.857
No	88(46.3%)	102(53.7%)	
Fear of death			
Yes	0	2(100%)	0.188
No	91(46.7%)	104(53.3%)	
Fear of divorce			
Yes	2(25%)	6(75%)	0.291
No	89(47.1%)	100(52.9%)	
Cultural beliefs			
Yes	11(28.2%)	28(71.8%)	0.021
No	80(50.6%)	78(49.4%)	
Unavailability of a female doctor			
Yes	16(42.1%)	22(57.9%)	0.574
No	75(47.2%)	84(52.8%)	
Financial constraints			
Yes	40(38.1%)	65(61.9%)	0.015
No	51(55.4%)	41(44.6%)	
Access to healthcare			
Yes	27(45.8%)	32(54.2%)	0.937
No	64(46.4%)	74(53.6%)	
Embarrassment			
Yes	18(41.9%)	25(58.1%)	0.519
No	73(47.4%)	81(52.6%)	
Lack of support			
Yes	35(40.7%)	51(59.3%)	0.173
No	56(50.5%)	55(49.5%)	
Quacks or herbal treatment			
Yes	16(42.1%)	22(57.9%)	0.672
No	73(45.9%)	86(54.1%)	
Health insurance			
Yes	1(33.3%)	2(66.7%)	0.99
No	90(46.4%)	104(53.6%)	
Cosmetic disfigurement			
Yes	6(54.5%)	5(45.5%)	0.757
No	85(45.7%)	101(54.3%)	

## Discussion

The morbidity and mortality rate of BC patients is directly associated with a delay in the treatment of patients [12]. This research was conducted to identify the factors behind the delay in the treatment of BC in our country. By understanding the reasons for delay it might be helpful in reducing the delay time and early detection of disease.

Patients with BC presenting late is the important contributing factor for treatment delay. Our findings correspond with the previous literature. A study by Maghous et al. found about 70% of the females presented late for the treatment due to personal reasons and 72% of the females with symptoms of BC had a delay of more than 6 months [11]. Talpur et al. in their study found approximately 95% of females took medical consultation after six months of diagnosis and 38% of the females presented with stage 3 of tumor [13]. In another similar study conducted by Gulzar et al. found 89% of the females presented late after a delay of more than three months for treatment [7]. Baig et al. observed in their study that means delay was 8.1 months and almost 66% of the patients were presented with a delay of more than six months [14]. 

In the present study, the mean of the patients was 45.38 years at the time of diagnosis. Almost the same result, the mean age of 44.1 years has been observed in the study in Pakistan by Gulzar et al. [7.] Whereas a study conducted in India showed a higher mean age of the patients such as 51.05 years at the time of diagnosis [15]. In the present study, 70.6% of the patients were living in urban areas and most of them were illiterate (52.3%) and belonging to the low class (75.6%). We also found that most of the females were married and housewives. A similar study conducted by Khan MA et al. also found that majority of the females were of age more than 40 years, had education less than 8 years, and belonged from poor and low socio-economic class and married [10]. Similar results were found by Gulzar et al. in their study [7]. In a Malaysian study by Norsaadah et al., most of the females were married and housewives, however education level is slightly more up to high school education among them [16]. In the present study, 70.6% of the females were from urban areas as this study was conducted at the tertiary care hospital of Karachi. In a study conducted at Rawalpindi and Islamabad about 69% of the females belonged from rural areas, 95% were married and approximately 82% had low-income levels [17]. 

In the present study, 53.3% of women had financial constraints followed by lack of support (43.7%), lack of awareness regarding treatment (30.5%), and inaccessibility to healthcare. The reason behind the high frequency of these factors is low-income level, illiteracy, and residence away from specialized healthcare. The majority of the Pakistani population has low-income levels due to which high fees of educational institutes are unaffordable for them. In the present study, many other factors such as cultural beliefs and prior use of traditional methods were identified as contributing factors for delay in treatment of BC due to limited education. The misconceptions that a person having cancer cannot survive and there is no treatment available are very common among the Pakistani populace. Therefore the lack of awareness and support are highly associated with treatment delay in patients with BC [18,19]. The study by Ayaz et al. reported 62.3% of the females presented late because they were unaware of the treatment of BC [17]. 

Gulzar et al. in their study found that 96% of the females presented late because they ignore the symptoms and painless lump in the breast, 81% had fear related to treatment expenses, 73% were shy to be treated by a male doctor, 71% had prior use of traditional methods, 65% had a social stigma, 61% were used to visit spiritual healers and 37% women had inaccessibility to healthcare [7]. A study by Shamsi et al. found patients presented late due to lack of awareness (58.1%), he also found 16.1% of the patients had embarrassment, misconceptions, fears, and shame related to the treatment of BC and 9.2% of the females were delay due to alternative medicine/ traditional method and had other reasons like financial constraints, family commitments and husband reaction to BC [20]. 

The social taboos such as embarrassment and shyness of females when discussing health-related concerns of the breast also causes a delay in treatment of BC. Most of them use alternative medicines (72%) for treatment, they don’t pay attention to indications of BC and seek medical advice from quacks or spiritual healers rather than oncologists [7,21]. In the present study factors such as social embarrassment regarding disease diagnosis in 21.8% of women, avoided treatment due to pardah (veil) among 19.3% women, appointment delay due to unavailability of consultant among 6.1% women, concern regarding cosmetic disfigurement among 5.6% women, anxiety related to divorce in 4.1% and surgery in 3.6% were observed. Other factors such as prior visits to quacks, no health insurance, and fear of death contributed a very less proportion in the delay of treatment of BC. Studies found that anxiety related to cancer treatment caused delays among 12% of women in developing countries [22,23]; specifically in patients with positive BC family history (82%). The wrong information regarding the adverse effects and chemotherapy toxicity directed to refusal and fear of treatment as well. Anxiety regarding separation or remarriage of the spouse also directed some women to take decisions against the treatment of BC. The belief that surgery or treatment causes disability and disfigurement is also related to the late presentation of BC [24].

There is a dire need for health education and health promotion among females of both rural and urban areas of Pakistan. The accessibility to healthcare should be improved which could increase the cure rate at the early stage of the tumor. The government and NGOs should also play a role by making policy for the treatment of BC and funds should be arranged for unaffordable patients. In the long term, these efforts will decrease the prevalence and increase the survival rate of BC patients in Pakistan. 

## Conclusions

The results of the present study have identified that cultural beliefs, poor financial status, appointment delay, and lack of awareness are the significant factors for the treatment delay in BC patients. There is a dire need for health education and health promotion among females of both rural and urban areas of Pakistan. The screening test should be made available and cost-effective for patients having low-income levels.

## References

[1] Parkin DM, Bray F, Ferlay J, Pisani P (2005). Global Cancer Statistics, 2002. CA Cancer J Clin.

[2] Menhas R, Umer S (2015). Breast cancer among Pakistani women. Iran J Public Health.

[3] Sohail S, Alam SN (2007). Breast cancer in Pakistan - awareness and early detection. J Coll Physicians Surg Pak.

[4] Ahmad Z, Idrees R, Fatima S (2016). Commonest Cancers in Pakistan - Findings and Histopathological Perspective from a Premier Surgical Pathology Center in Pakistan. Asian Pac J Cancer Prev.

[5] Romeiro Lopes TC, Gravena AAF, Demitto MO (2017). Delay in diagnosis and treatment of breast cancer among women attending a reference service in Brazil. Asian Pac J Cancer Prev.

[6] Al-Amri AM (2015). Clinical presentation and causes of the delayed diagnosis of breast cancer in patients with pregnancy associated breast cancer. J Family Community Med.

[7] Gulzar F, Akhtar MS, Sadiq R, Bashir S, Jamil S, Baig SM (2019). Identifying the reasons for delayed presentation of Pakistani breast cancer patients at a tertiary care hospital. Cancer Manag Res.

[8] Aziz Z, Sana S, Akram M, Saeed A (2004). Socioeconomic status and breast cancer survival in Pakistani women. J Pak Med Assoc.

[9] Majeed AI, Jadoon M, Riazuddin S, Akram J (2017). Awareness and screening of breast cancer among rural areas of Islamabad capital territory, Pakistan. Ann PIMS.

[10] Khokher S, Qureshi MU, Mahmood S, Sadiq S (2016). Determinants of advanced stage at initial diagnosis of breast cancer in Pakistan: adverse tumor biology vs delay in diagnosis. Asian Pac J Cancer Prev.

[11] Maghous A, Rais F, Ahid S (2016). Factors influencing diagnosis delay of advanced breast cancer in Moroccan women. BMC Cancer.

[12] Smith EC, Ziogas A, Anton-Culver H (2013). Delay in surgical treatment and survival after breast cancer diagnosis in young women by race/ethnicity. JAMA Surg.

[13] Talpur AA, Surahio AR, Ansari A, Ghumro AA (2011). Late presentation of breast cancer: a dilemma. J Pak Med Assoc.

[14] Baig M, Sohail I, Altaf HN, Altaf OS (2019). Factors influencing delayed presentation of breast cancer at a tertiary care hospital in Pakistan. Cancer Rep.

[15] Tiwari V, Yogi V, Ghori HU (2015). Identifying the factors causing delayed presentation of cancer patients to a Government Medical College of Central India. J Clin Diagn Res.

[16] Norsa'adah B, Rampal KG, Rahmah MA, Naing NN, Biswal BM (2011). Diagnosis delay of breast cancer and its associated factors in Malaysian women. BMC Cancer.

[17] Ogunkorode RS, Holtslander L, Ferguson L, Maree JE, Anonson J, Ramsden VR (2021). Factors influencing the health-seeking behaviors of women with advanced stages of breast cancer in Southwestern Nigeria: an interpretive description study.. Int J Afr Nurs Sci.

[18] Khan MA, Hanif S, Iqbal S, Shahzad MF, Shafique S, Khan MT (2015). Presentation delay in breast cancer patients and its association with sociodemographic factors in North Pakistan. Chin J Cancer Res.

[19] Opoku SY, Benwell M, Yarney J (2012). Knowledge, attitudes, beliefs, behaviour and breast cancer screening practices in Ghana, West Africa. Pan Afr Med J.

[20] Shamsi U, Khan S, Azam I (2020). Patient delay in breast cancer diagnosis in two hospitals in Karachi, Pakistan: preventive and life-saving measures needed. JCO Glob Oncol.

[21] Khan MA, Ahmed M, Ahmed N (2017). Treatment navigation pathway and barriers to treatment for cancer patients in Khyber Pakhtunkhwa, Pakistan. J Med Sci.

[22] Landolsi A, Gahbiche S, Chaafii R (2010). Reasons of diagnostic delay of breast cancer in Tunisian women (160 patients in the central region of Tunisia). Tunis Med.

[23] Ermiah E, Abdalla F, Buhmeida A, Larbesh E, Pyrhönen S, Collan Y (2012). Diagnosis delay in Libyan female breast cancer. BMC Res Notes.

[24] Grunfeld EA, Hunter MS, Ramirez AJ, Richards MA (2003). Perceptions of breast cancer across the lifespan. J Psychosom Res.

